# Is there a tape recorder in your head? How the brain stores and retrieves musical melodies

**DOI:** 10.3389/fnsys.2014.00149

**Published:** 2014-08-28

**Authors:** Josef P. Rauschecker

**Affiliations:** ^1^Department of Neuroscience, Georgetown University Medical CenterWashington, DC, USA; ^2^Institute for Advanced Studies, Technical University MunichGarching, Germany

**Keywords:** auditory dorsal stream, premotor cortex, basal ganglia, temporal combination sensitivity, sound sequence, auditory object, auditory ventral stream, prefrontal cortex

## Abstract

Music consists of strings of sound that vary over time. Technical devices, such as tape recorders, store musical melodies by transcribing event times of temporal sequences into consecutive locations on the storage medium. Playback occurs by reading out the stored information in the same sequence. However, it is unclear how the brain stores and retrieves auditory sequences. Neurons in the anterior lateral belt of auditory cortex are sensitive to the combination of sound features in time, but the integration time of these neurons is not sufficient to store longer sequences that stretch over several seconds, minutes or more. Functional imaging studies in humans provide evidence that music is stored instead within the auditory dorsal stream, including premotor and prefrontal areas. In monkeys, these areas are the substrate for learning of motor sequences. It appears, therefore, that the auditory dorsal stream transforms musical into motor sequence information and vice versa, realizing what are known as forward and inverse models. The basal ganglia and the cerebellum are involved in setting up the sensorimotor associations, translating timing information into spatial codes and back again.

## Musical melodies as sequences and objects

Musical melodies are sequences of sound with particular rhythm, loudness and timbre. As such, they are concatenations of discrete elements over time, which can continue for seconds or minutes. However, we can learn to recognize melodies as a single entity, as we recognize extended objects in either the visual or auditory modality, and we can assign a name to them (“Twinkle, twinkle, little star” or “Yankee doodle”). In this more holistic view, a melody is an entity that requires integration of its elements over time and, ultimately, coding by a specific, limited ensemble of neurons in the brain. This latter representation is likely to be situated in the auditory ventral stream, where representations of “auditory objects” have been found (Tian et al., 2001; Zatorre et al., 2004). In a hierarchical model, information about spectral structure and temporal modulation, including pitch, are stored in early ventral areas and in core (Leaver and Rauschecker, 2010; Schindler et al., 2013); higher-order object information, e.g., about timbre, which would reveal the identity of an instrument or singer, is most likely found in the anterior-most regions of superior temporal cortex (Leaver and Rauschecker, 2010) and in ventrolateral prefrontal cortex (Cohen et al., 2009; Plakke et al., 2013). Even in the most hierarchical model, however, it seems unlikely to find single neurons responding selectively to lengthy melodies, just as it seems unreasonable to expect single neurons to respond to specific sentences in the language domain. So how is the identity of a sound sequence warranted in the brain?

For speech, regions in the anterior superior temporal cortex (aSTC) have been found that respond to phonemes or words, including short standard phrases (DeWitt and Rauschecker, 2012), but not to whole sentences. The latter would seem to reside in the auditory dorsal stream instead, where representations of sequences have been found (Schubotz et al., 2004). Activation of dorsal-stream regions, including supplementary and pre-supplementary motor areas (SMA, pre-SMA) or ventral and dorsal premotor cortex (vPMC, dPMC), has also been reported during singing (Perry et al., 1999), listening to music (Chen et al., 2008), and during anticipatory imagery of music (Leaver et al., 2009).

But how does the storage process of lengthy sound sequences really happen? This is not at all a trivial question, and the brain mechanisms governing the processing, storage and retrieval of sequences are far from understood. It may be advantageous, therefore, to briefly consider how technical devices do this.

## How tape recorders work

A tape recorder is an audio storage device that records and plays back sounds, including music and speech, using magnetic tape as a storage medium. It records a fluctuating audio signal by moving the tape across a “tape-head” that polarizes the magnetic domains in the tape in proportion to the audio signal (modified from Wikipedia). Electric current flowing in the tape-head creates a fluctuating magnetic field, which causes the magnetic material on the tape to align in a manner proportional to the original signal, as the tape is moving past the head. The original signal can be re-produced by running the tape back across the tape head, where the reverse process occurs—the magnetic imprint on the tape induces a small current in the reading head, which approximates the original signal and is then amplified for playback on a loudspeaker (from Wikipedia).

Thus, a tape recorder stores music by moving a storage medium (the tape) past a device (the head) that represents the sound waves in the form of a fluctuating electro-magnetic field. A turntable or CD player follows the same principle of using the movement of a recording medium to translate time into space, this time in the form of a spiral track. In all cases, the recording process can be inverted into a playback process by the reverse mechanism, moving the recorded medium past the reading device at the same speed, thus recreating the original signal.

The important message to be gleaned from this is that technical devices store musical melodies (as well as other sequences) by re-coding time of occurrence into spatial positions. Furthermore, storage and retrieval of the sequence utilize the same mechanism, differing only by inversion. Applied to the brain, it is attractive to think that information is stored in the same places where the original activation takes place, and that recording and read-out are also accomplished by similar, but inverse mechanisms. But how is the order of events in a time sequence preserved? At first, the only way to form temporal associations between stored items would seem to be by “chaining” the events together, whereby one event becomes the cue for the next one (Ebbinghaus, 1964). Read-out takes the form of cued recall. Although this idea has been criticized (Lashley, 1951; Terrace, 2005), it still provides one possible mechanism for storing a sequence, but it remains unclear how it is implemented in the brain.

Obviously, unlike a tape recorder or CD player, the brain does not have any moving parts for the translation of time into space. Then again, digital storage devices (solid-state or flash drives) no longer require moving tapes or spinning discs. These devices store audio as a stream of numbers representing the amplitude of the audio signal at equal time intervals. The numbers get stored in the order they are received, and a “controller” assures that they are read out in the same order later. This form of storing a sequence requires a positional code, i.e., the re-coding of event time into position in space, something that has been postulated variously for models of short-term memory as well (Henson and Burgess, 1997).

In summary, technical devices universally store sequences by re-coding time of occurrence into spatial positions, and the fundamental question arises: How *does* the brain translate temporal events in a sequence into spatial patterns or a spatial gradient?

## Neural mechanisms for the encoding of sequential order

### Temporal combination sensitivity as the most elementary mechanism

Most simplistically, music consists of two essential elements: frequency (or pitch) and rhythm. However, while rhythm (duration of tones and the intervals between them) is obviously important, we can still recognize a melody (within limits) even when rhythmic elements are omitted. Recent results confirm that pitch and rhythm are indeed processed and stored independently (Schellenberg et al., 2014). Thus, the most essential element for the recognition of a melody is the order of the notes it consists of. If that order is changed, or the melody is played in reverse, recognition is impaired or fails altogether. Again, there is commonality between music and language (c.f. Patel, 2008; Patel and Iversen, 2014), as language comprehension also becomes impossible when its elements are played in reverse (either at the word or sentence level) (Bornkessel-Schlesewsky and Schlesewsky, 2013).

**Figure 1:**
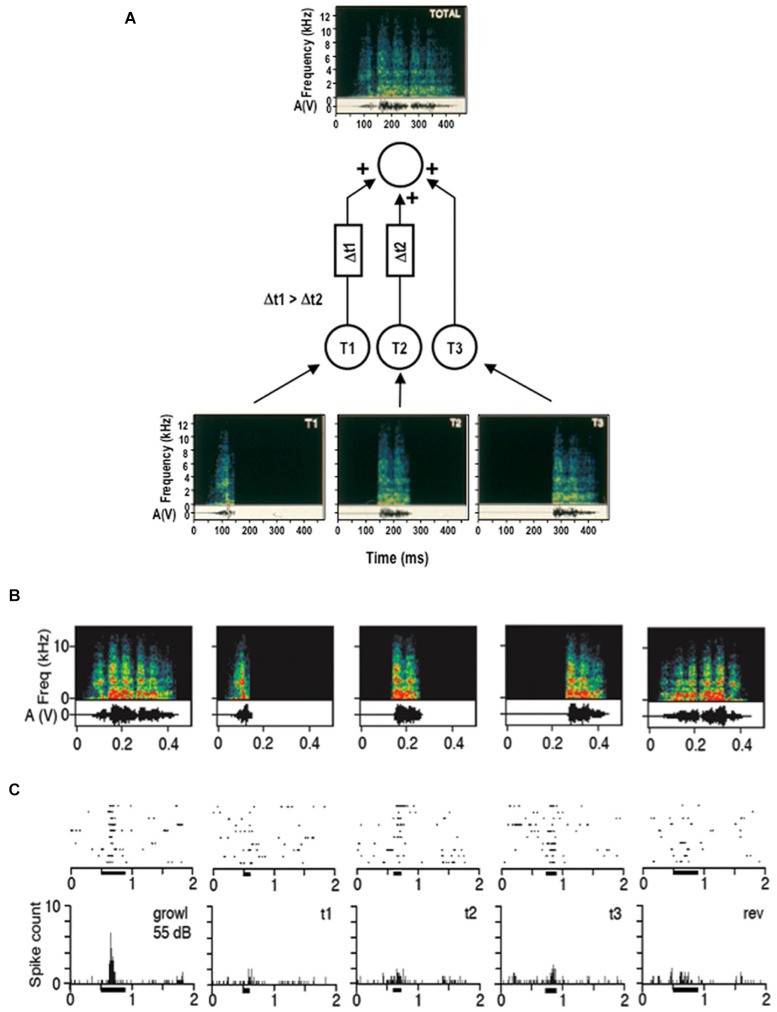
**Auditory direction selectivity of a cortical neuron as cellular basis for sequence selectivity. (A)** Schematic drawing of a neuron in the lateral belt of rhesus monkey auditory cortex, illustrating temporal combination sensitivity (TCS). Input from lower-order neurons is integrated at the level of the lateral belt in a nonlinear fashion (Rauschecker et al., 1995). The belt neuron acts as a logical AND-gate and fires only if the membrane potential surpasses a given threshold. Temporal delay lines generate order sensitivity such that a sound sequence will excite the neuron only if presented in a specific order (from Rauschecker, 2012). **(B, C)** Example of a response by a neuron in the lateral belt to a species-specific vocalization. Spectrograms of the call and its temporal components are shown in **(B)** together with the reversed call (on extreme right). The neuron’s response (shown in **(C)**) to individual “syllables” and to the reversed call is strongly diminished.

A neural mechanism that is commonly invoked for implementing this reversal sensitivity is the combination of inputs over time (temporal combination sensitivity, TCS). Just as in its twin mechanism, spectral combination sensitivity (Margoliash and Fortune, 1992), the target neuron acts as a logical AND-gate which fires only if several inputs are active simultaneously. In the time domain, delay lines can be used to hold up some of the inputs long enough until all other inputs have arrived (Figure 1). These asymmetric delays have the effect of creating selectivity for temporal order on a short time scale in the order of hundreds of milliseconds. Thus, temporally asymmetric delays can be created by spatial asymmetries on a miniature scale similar to direction selectivity in the visual system. This mechanism creates FM detectors with pronounced selectivity for the direction of an FM sweep (Tian and Rauschecker, 2004; see also Tian et al., 2013 for further analogies between elemental detectors in visual and auditory cortex).

### Premotor areas as sequencing machines

While the above TCS mechanism works well at durations corresponding to syllable or word level, it breaks down when the strings of sound become longer. Under those circumstances, one may assume that chaining mechanisms come into play, where the end of one short sequence triggers the beginning of the next, like in a game of dominoes. Such mechanisms have been postulated in particular for the motor system, where the execution of smooth movements requires precise timing and order of muscle activations. Brain substrates that play a role for the learning, planning and execution of such sequential behavior are thought to be the cerebellum, the striatum, and various regions of premotor and prefrontal cortex (Hikosaka et al., 1996; Sakai et al., 1999; Fuster et al., 2000; Yin, 2010). While premotor and prefrontal areas are most important for planning and execution, cerebellum and basal ganglia are involved at different stages of learning of a motor sequence. In particular, cerebellum and striatum differ by the time scales they apply to the transformation of temporal into spatial patterns.

It is important to keep in mind that music is often created by another person making it. That is, someone is producing the music before we can listen to it, and a melody is first and foremost a motor sequence that happens to produce sounds. This is true even if we produce the music ourselves. We produce music by virtue of activating muscles that move our vocal cords, lips and jaws (during singing or whistling) or, depending on the type of musical instrument played, we move our arms, fingers, feet, and sometimes our lips in coordination with our breathing apparatus (This is similar again in speech, where we learn to produce a sound by moving our muscles of the lips, tongue etc. in coordination with the vocal cords and breathing muscles). Hearing another person produce these sounds may trigger the same or similar muscle movements, with the goal of producing the same sounds. This may happen either as a form of imitation, or directly as a result of sensorimotor interaction that, by necessity, intertwines perception and action during the production of these sounds. In other words, the feedback from hearing (and to some extent proprioception) is a necessary prerequisite for normal production of producible sounds. The process can best be appreciated in reference to speaking or singing, where we have the same “instrument”, our vocal apparatus, at our disposal as the models we are trying to emulate. However, even when listening to a musical instrument that we are not capable of playing ourselves, we can produce the same melody by generating tones in the same order and with the same timing as the ones we listen to.

It will be interesting to find out when this ability to re-produce sound sequences first develops. Although young infants have the ability to recognize familiar melodies as early as 2 months of age, they do not develop relative pitch until ~6 months of age and not without exposure to music (Plantinga and Trainor, 2009).

### Sensorimotor learning in nonhuman primates

**Figure 2:**
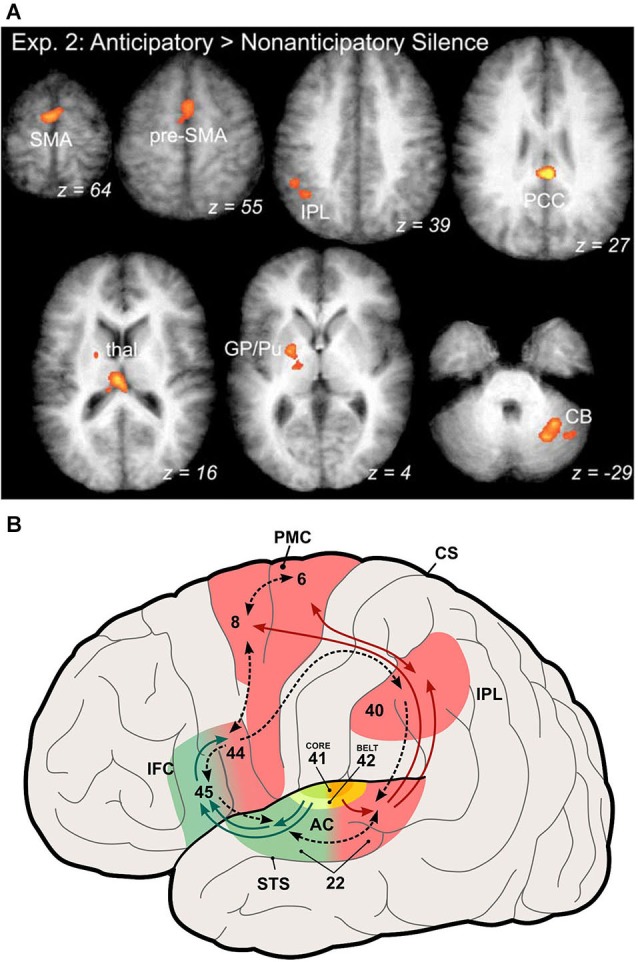
**Participation of auditory dorsal stream in coding of musical sequences. (A)** Activation of areas in the auditory dorsal stream by anticipation of familiar music. Activated areas include the supplementary and pre-supplementary motor areas (SMA, pre-SMA), the inferior parietal lobule (IPL), posterior cingulate cortex (PCC), globus pallidus and putamen (GP/Pu) of the basal ganglia, and the cerebellum (CB) (from Leaver et al., 2009). **(B)** Illustration of the auditory ventral and dorsal streams in the human brain (modified from Rauschecker and Scott, 2009). This expanded model originated from the original dual-pathway model of Rauschecker and Tian (2000) by generalizing the role of the dorsal stream to one of sensorimotor integration and control, which includes processing of space and motion as well as storage and retrieval of sound sequences, the latter especially relevant for processing of music.

Studies in monkeys have shown that during learning of a new sensorimotor association the basal ganglia are very active (Pasupathy and Miller, 2005). The same has been shown by functional imaging studies in humans that are learning new sequences (Leaver et al., 2009; Yin, 2010). These results assign a role to the basal ganglia in the chaining or stitching together of new sensorimotor associations or, more succinctly, in the transformation of temporal order information into a spatial code (Kalm and Norris, 2014). After a sequence is well learned, activation of premotor and prefrontal regions becomes increasingly prominent, while basal ganglia activation weakens (Figure 2A; Leaver et al., 2009). This reflects the formation of chunks of sequence items, consistent with human learning and imaging studies (Janata and Grafton, 2003), which are stored in frontal areas like pre-SMA and SFG (Sakai et al., 1999; Sakai and Passingham, 2003). The activation moves more rostral as the sequence becomes more familiar (Leaver et al., 2009). This is consistent with a caudal-to-rostral hierarchy within prefrontal cortex (Badre and D’Esposito, 2009), where rostral areas control activity in more caudal modality-specific areas (Sakai and Passingham, 2003).

It is currently unclear if it is possible to learn a new melody or sequence without engaging these sensorimotor mechanisms by just passively listening to it. As a melody becomes increasingly familiar, it often becomes impossible to suppress the urge to sing along. While the learning of a new song or a new piece played on an instrument results in the building of “muscle memory” by tuning the motor and premotor structures of the brain, this may not happen in individuals that lack the corresponding skills. It would be interesting to see if there are certain forms of amusia that lack the ability to reproduce or recognize music, and whether this is actually a weakness of their sensorimotor memory and also affects their general ability to remember sequences (c.f. Tremblay-Champoux et al., 2010). Interestingly, some forms of congenital amusia involve structural changes in the inferior frontal region (Hyde et al., 2007), but more research is needed to possibly tie these changes to a domain-general deficit in sequence processing.

### Singing in birds

Vocal learning is not unique to humans. It is common in a variety of animal species (Patel and Iversen, 2014), especially birds. Some songbird species (such as zebra finches or starlings) learn their melodies from a conspecific teacher, usually their father (Comins and Gentner, 2010; Adret et al., 2012); others (such as parrots or bullfinches) can also imitate words or melodies they hear from humans (Eda-Fujiwara et al., 2012; Nicolai et al., 2014).

A wealth of neurobiological studies in several songbird species suggests that their neural apparatus for audio-motor learning is quite similar in principle to that of humans and nonhuman primates, consisting of premotor-basal-ganglia circuits that work in conjunction with higher auditory centers to encode the memorized songs (Achiro and Bottjer, 2013). In particular, recent data from zebra finches show that vocal motor circuits also participate in the encoding of auditory experience of the vocal model (Roberts and Mooney, 2013). Thus, a universal circuit model is beginning to emerge from these comparative studies that might ultimately lead to an understanding of storage and retrieval of sound sequences in biological systems.

## Synthesis: melodies in ventral and dorsal streams

Much evidence suggests that the dual auditory processing streams originally postulated for the monkey (Rauschecker, 1997, 1998; Romanski et al., 1999; Rauschecker and Tian, 2000) also exist in humans. The ventral auditory stream is important for the encoding of complex spectral information, including pitch (Bendor and Wang, 2005), and ultimately for the identification of sound objects. The dorsal stream was originally defined by its involvement in auditory spatial processing (Rauschecker and Tian, 2000) and movement in space (Warren et al., 2002). This is still believed to be correct (Rauschecker, 2012), but the role of the dorsal stream has been expanded to include sensorimotor integration and control in more general terms (Rauschecker and Scott, 2009; Rauschecker, 2011), including the representation of sequences.

A particularly interesting and important feature of the expanded dorsal stream is that it represents both inverse and forward models (Figure 2B). The forward model is what has classically been referred to as an “efference copy” (von Holst and Mittelstaedt, 1950; Troyer and Doupe, 2000). Whenever premotor cortex neurons fire in preparation of an action, they not only send their message towards the motor cortex for potentially real action, but they also inform sensory systems about the consequences of this action. Conversely, an inverse model (Grush, 2004) instructs the motor system about sensory signals that are relevant for reaching its goals. Both of these signals are compared within the dorsal stream, presumably in parietal cortex, and play a role for optimal state estimation by minimizing the resulting error signal (Rauschecker and Scott, 2009).

The ability of posterior parietal cortex to perform transformations in space may also come to bear in terms of melodic “space”. We can easily recognize a melody when it is played in a different key, that is, when pitch relations between notes are preserved. An imaging study contrasting a transposed melody to the original melody revealed greater activation in the intraparietal sulcus (IPS; Foster and Zatorre, 2010), which points to the role of the IPS in subtracting the effects of the transposition.

Finally, the question arises whether musical melodies, once they are learned, are simply defined by their existence as concatenated sequences in sensorimotor regions of the auditory dorsal stream. The fact that they can be sung or played, imagined and anticipated almost automatically on a given cue seems to demonstrate that this is indeed the case. However, as mentioned in the Introduction section, we can also put a name or a label on a familiar melody, which suggests that there is a second form of existence for music in the brain besides concatenated sounds. The “chunks” formed in rostral prefrontal cortex that become apparent in fMRI studies of highly familiar music, may be the endpoint of the sequencing process in the dorsal stream. At the same time, however, they may also be the starting point of a feedback process (via the inferior frontal cortex) into the ventral auditory pathway, where more information is added, for instance, about the timbre of musical instruments playing a specific tune or about its emotional connotations. This object-identification process would enable a musical melody not just to receive a name, but also to trigger memories of all things past that are associated with that melody.

## Conflict of interest statement

The author declares that the research was conducted in the absence of any commercial or financial relationships that could be construed as a potential conflict of interest.

## References

[B1] Achiro J. M. Bottjer S. W. (2013). Neural representation of a target auditory memory in a cortico-basal ganglia pathway. J. Neurosci. 33, 14475–14488 10.1523/JNEUROSCI.0710-13.2013 24005299 PMC3761053

[B2] Adret P. Meliza C. D. Margoliash D. (2012). Song tutoring in presinging zebra finch juveniles biases a small population of higher-order song-selective neurons toward the tutor song. J. Neurophysiol. 108, 1977–1987 10.1152/jn.00905.2011 22786956 PMC3544995

[B3] Badre D. D’Esposito M. (2009). Is the rostro-caudal axis of the frontal lobe hierarchical? Nat. Rev. Neurosci. 10, 659–669 10.1038/nrn2667 19672274 PMC3258028

[B4] Bendor D. Wang X. (2005). The neuronal representation of pitch in primate auditory cortex. Nature 436, 1161–1165 10.1038/nature03867 16121182 PMC1780171

[B5] Bornkessel-Schlesewsky I. Schlesewsky M. (2013). Reconciling time, space and function: a new dorsal-ventral stream model of sentence comprehension. Brain Lang. 125, 60–76 10.1016/j.bandl.2013.01.010 23454075

[B6] Chen J. L. Penhune V. B. Zatorre R. J. (2008). Moving on time: brain network for auditory-motor synchronization is modulated by rhythm complexity and musical training. J. Cogn. Neurosci. 20, 226–239 10.1162/jocn.2008.20018 18275331

[B7] Cohen Y. E. Russ B. E. Davis S. J. Baker A. E. Ackelson A. L. Nitecki R. (2009). A functional role for the ventrolateral prefrontal cortex in non-spatial auditory cognition. Proc. Natl. Acad. Sci. U S A 106, 20045–20050 10.1073/pnas.0907248106 19897723 PMC2785289

[B8] Comins J. A. Gentner T. Q. (2010). Working memory for patterned sequences of auditory objects in a songbird. Cognition 117, 38–53 10.1016/j.cognition.2010.06.009 20638052 PMC2934891

[B9] DeWitt I. Rauschecker J. P. (2012). Phoneme and word recognition in the auditory ventral stream. Proc. Natl. Acad. Sci. U S A 109, E505–E514 10.1073/pnas.1113427109 22308358 PMC3286918

[B10] Ebbinghaus H. (1964). Memory: A Contribution to Experimental Psychology. (New York: Dover) (Original work published as: Ebbinghaus, H. (1885). Über das Gedächtnis. Leipzig, Germany: Duncker and Humblot).

[B11] Eda-Fujiwara H. Imagawa T. Matsushita M. Matsuda Y. Takeuchi H. A. Satoh R. (2012). Localized brain activation related to the strength of auditory learning in a parrot. PLoS One 7:e38803 10.1371/journal.pone.0038803 22701714 PMC3372503

[B12] Foster N. E. Zatorre R. J. (2010). A role for the intraparietal sulcus in transforming musical pitch information. Cereb. Cortex 20, 1350–1359 10.1093/cercor/bhp199 19789184

[B13] Fuster J. M. Bodner M. Kroger J. K. (2000). Cross-modal and cross-temporal association in neurons of frontal cortex. Nature 405, 347–351 10.1038/35012613 10830963

[B14] Grush R. (2004). The emulation theory of representation: motor control, imagery and perception. Behav. Brain Sci. 27, 377–396; discussion 396–442 10.1017/s0140525x04000093 15736871

[B15] Henson R. Burgess N. (1997). “Representations of serial order,” in 4th Neural Computation and Psychology Workshop, eds Bullinaria J. A. Glasspool D. W. Houghton G. (London: Springer), 283–300

[B16] Hikosaka O. Sakai K. Miyauchi S. Takino R. Sasaki Y. Pütz B. (1996). Activation of human presupplementary motor area in learning of sequential procedures: a functional MRI study. J. Neurophysiol. 76, 617–621 8836248 10.1152/jn.1996.76.1.617

[B17] Hyde K. L. Lerch J. P. Zatorre R. J. Griffiths T. D. Evans A. C. Peretz I. (2007). Cortical thickness in congenital amusia: when less is better than more. J. Neurosci. 27, 13028–13032 10.1523/jneurosci.3039-07.2007 18032676 PMC6673307

[B18] Janata P. Grafton S. T. (2003). Swinging in the brain: shared neural substrates for behaviors related to sequencing and music. Nat. Neurosci. 6, 682–687 10.1038/nn1081 12830159

[B19] Kalm K. Norris D. (2014). The representation of order information in auditory-verbal short-term memory. J. Neurosci. 34, 6879–6886 10.1523/JNEUROSCI.4104-13.2014 24828642 PMC4019801

[B20] Lashley K. (1951). “The problem of serial order in behavior,” in Cerebral Mechanisms in Behavior, ed Jeffress L. (Oxford: Wiley and Sons), 112–136

[B52] Leaver A. M. Rauschecker J. P. (2010). Cortical representation of natural complex sounds: effects of acoustic features and auditory object category. J. Neurosci. 30, 7604–7612 10.1523/JNEUROSCI.0296-10.2010 20519535 PMC2930617

[B21] Leaver A. M. Van Lare J. Zielinski B. Halpern A. R. Rauschecker J. P. (2009). Brain activation during anticipation of sound sequences. J. Neurosci. 29, 2477–2485 10.1523/JNEUROSCI.4921-08.2009 19244522 PMC2892726

[B22] Margoliash D. Fortune E. S. (1992). Temporal and harmonic combination-sensitive neurons in the zebra finch’s HVc. J. Neurosci. 12, 4309–4326 1432096 10.1523/JNEUROSCI.12-11-04309.1992 PMC6575994

[B23] Nicolai J. Gundacker C. Teeselink K. Güttinger H. R. (2014). Human melody singing by bullfinches (Pyrrhula pyrrula) gives hints about a cognitive note sequence processing. Anim. Cogn. 17, 143–155 10.1007/s10071-013-0647-6 23783267

[B24] Pasupathy A. Miller E. K. (2005). Different time courses of learning-related activity in the prefrontal cortex and striatum. Nature 433, 873–876 10.1038/nature03287 15729344

[B25] Patel A. D. (2008). Music, Language and the Brain. New York: Oxford University Press

[B26] Patel A. D. Iversen J. R. (2014). The evolutionary neuroscience of musical beat perception: the Action Simulation for Auditory Prediction (ASAP) hypothesis. Front. Syst. Neurosci. 8:57 10.3389/fnsys.2014.00057 24860439 PMC4026735

[B27] Perry D. W. Zatorre R. J. Petrides M. Alivisatos B. Meyer E. Evans A. C. (1999). Localization of cerebral activity during simple singing. Neuroreport 10, 3979–3984 10.1097/00001756-199911080-00035 10716244

[B28] Plakke B. Diltz M. D. Romanski L. M. (2013). Coding of vocalizations by single neurons in ventrolateral prefrontal cortex. Hear. Res. 305, 135–143 10.1016/j.heares.2013.07.011 23895874 PMC3979279

[B29] Plantinga J. Trainor L. J. (2009). Melody recognition by two-month-old infants. J. Acoust. Soc. Am. 125, EL58–EL62 10.1121/1.3049583 19206833

[B30] Rauschecker J. P. (1997). Processing of complex sounds in the auditory cortex of cat, monkey and man. Acta Otolaryngol. Suppl. 532, 34–38 10.3109/00016489709126142 9442842

[B31] Rauschecker J. P. (1998). Cortical processing of complex sounds. Curr. Opin. Neurobiol. 8, 516–521 10.1016/s0959-4388(98)80040-8 9751652

[B54] Rauschecker J. P. (2011). An expanded role for the dorsal auditory pathway in sensorimotor integration and control. Hear. Res. 271, 16–25 10.1016/j.heares.2010.09.001 20850511 PMC3021714

[B32] Rauschecker J. P. (2012). “Processing streams in the auditory cortex,” in Neural Correlates of Auditory Cognition, eds Cohen Y. E. Fay R. R. Popper A. N. (New York, Heidelberg: Springer Handbook of Auditory Research), 7–44

[B33] Rauschecker J. P. Scott S. K. (2009). Maps and streams in the auditory cortex: non-human primates illuminate human speech processing. Nat. Neurosci. 12, 718–724 10.1038/nn.2331 19471271 PMC2846110

[B34] Rauschecker J. P. Tian B. (2000). Mechanisms and streams for processing of “what” and “where” in auditory cortex. Proc. Natl. Acad. Sci. U S A 97, 11800–11806 10.1073/pnas.97.22.11800 11050212 PMC34352

[B35] Rauschecker J. P. Tian B. Hauser M. (1995). Processing of complex sounds in the macaque nonprimary auditory cortex. Science 268, 111–114 10.1126/science.7701330 7701330

[B36] Roberts T. F. Mooney R. (2013). Motor circuits help encode auditory memories of vocal models used to guide vocal learning. Hear. Res. 303, 48–57 10.1016/j.heares.2013.01.009 23353871 PMC3689868

[B37] Romanski L. M. Tian B. Fritz J. Mishkin M. Goldman-Rakic P. S. Rauschecker J. P. (1999). Dual streams of auditory afferents target multiple domains in the primate prefrontal cortex. Nat. Neurosci. 2, 1131–1136 10.1038/16056 10570492 PMC2778291

[B38] Sakai K. Hikosaka O. Miyauchi S. Sasaki Y. Fujimaki N. Pütz B. (1999). Presupplementary motor area activation during sequence learning reflects visuo-motor association. J. Neurosci. 19, RC1 10234047 10.1523/JNEUROSCI.19-10-j0002.1999 PMC6782738

[B39] Sakai K. Passingham R. E. (2003). Prefrontal interactions reflect future task operations. Nat. Neurosci. 6, 75–81 10.1038/nn987 12469132

[B40] Schellenberg E. G. Stalinski S. M. Marks B. M. (2014). Memory for surface features of unfamiliar melodies: independent effects of changes in pitch and tempo. Psychol. Res. 78, 84–95 10.1007/s00426-013-0483-y 23385775

[B41] Schindler A. Herdener M. Bartels A. (2013). Coding of melodic gestalt in human auditory cortex. Cereb. Cortex 23, 2987–2993 10.1093/cercor/bhs289 22989579 PMC3827712

[B42] Schubotz R. I. Sakreida K. Tittgemeyer M. von Cramon D. Y. (2004). Motor areas beyond motor performance: deficits in serial prediction following ventrolateral premotor lesions. Neuropsychology 18, 638–645 10.1037/0894-4105.18.4.638 15506831

[B43] Terrace H. S. (2005). The simultaneous chain: a new approach to serial learning. Trends Cogn. Sci. 9, 202–210 10.1016/j.tics.2005.02.003 15808503

[B44] Tian B. Kusmierek P. Rauschecker J. P. (2013). Analogues of simple and complex cells in the auditory cortex of the rhesus monkey. Proc. Natl. Acad. Sci. U S A 110, 7892–7897 10.1073/pnas.1221062110 23610391 PMC3651431

[B45] Tian B. Rauschecker J. P. (2004). Processing of frequency-modulated sounds in the lateral auditory belt cortex of the rhesus monkey. J. Neurophysiol. 92, 2993–3013 10.1152/jn.00472.2003 15486426

[B46] Tian B. Reser D. Durham A. Kustov A. Rauschecker J. P. (2001). Functional specialization in rhesus monkey auditory cortex. Science 292, 290–293 10.1126/science.1058911 11303104

[B47] Tremblay-Champoux A. Dalla Bella S. Phillips-Silver J. Lebrun M.-A. Peretz I. (2010). Singing proficiency in congenital amusia: imitation helps. Cogn. Neuropsychol. 27, 463–476 10.1080/02643294.2011.567258 21864199

[B48] Troyer T. W. Doupe A. J. (2000). An associational model of birdsong sensorimotor learning I. Efference copy and the learning of song syllables. J. Neurophysiol. 84, 1204–1223 10979996 10.1152/jn.2000.84.3.1204

[B49] von Holst E. Mittelstaedt H. (1950). Das Reafferenzprinzip: wechselwirkungen zwischen zentralnervensystem und peripherie. Naturwissenschaften 37, 464–476 10.1007/bf00622503

[B53] Warren J. D. Zielinski B. A. Green G. G. R. Rauschecker J. P. Griffiths T. D. (2002). Analysis of sound source motion by the human brain. Neuron 34, 1–20 11931748 10.1016/s0896-6273(02)00637-2

[B50] Yin H. H. (2010). The sensorimotor striatum is necessary for serial order learning. J. Neurosci. 30, 14719–14723 10.1523/JNEUROSCI.3989-10.2010 21048130 PMC3181000

[B51] Zatorre R. J. Bouffard M. Belin P. (2004). Sensitivity to auditory object features in human temporal neocortex. J. Neurosci. 24, 3637–3642 10.1523/jneurosci.5458-03.2004 15071112 PMC6729744

